# Neurodevelopment of Children Born with Forceps Delivery—A Single Tertiary Clinic Study

**DOI:** 10.3390/medicina60111743

**Published:** 2024-10-24

**Authors:** Sanja Kostic, Katarina Ivanovic, Ivana Jovanovic, Milos Petronijevic, Natasa Cerovac, Jelena Milin-Lazovic, Danijela Bratic, Stefan Dugalic, Miroslava Gojnic, Milica Petronijevic, Milan Stojanovic, Ivan Rankovic, Svetlana Vrzic Petronijevic

**Affiliations:** 1 Clinic for Gynecology and Obstetrics, University Clinical Center of Serbia, 11000 Belgrade, Serbia; cara.kostic@gmail.com (S.K.); ikatarina.1996@gmail.com (K.I.); ivana.jovanovic92@yahoo.com (I.J.); ordinacija.petronijevic@gmail.com (M.P.); danijelabratic72@gmail.com (D.B.); stef.dugalic@gmail.com (S.D.); miroslavagojnicdugalic@yahoo.com (M.G.); 2 Faculty of Medicine, University of Belgrade, 11000 Belgrade, Serbia; natasa.cerovac.npk@gmail.com (N.C.); milica.petronijevic05@gmail.com (M.P.); 3 Clinic for Neurology and Psychiatry for Children and Youth, University Clinical Center of Serbia, 11000 Belgrade, Serbia; 4 Faculty of Medicine, Institute for Medical Statistics and Informatics, University of Belgrade, 11000 Belgrade, Serbia; milinjelena@gmail.com; 5 Clinical Center Dr Dragisa Misovic, Hospital for Gynecology and Obstetrics, 11000 Belgrade, Serbia; 6 Department of Gastroenterology and Liver Unit, Royal Cornwall Hospitals NHS Trust, Truro TR1 3LJ, UK

**Keywords:** forceps, assisted vaginal birth, cesarean delivery, neurodevelopment

## Abstract

*Background and Objectives:* Forceps delivery is a crucial obstetrical technique that has become increasingly underutilized in favor of cesarean delivery, despite the numerous complications related to cesarean sections. The major concerns with regard to assisted vaginal birth (AVB) are safety and long-term consequences. We aimed to investigate a neurological outcome of neonates and children at the age of 7 who were born via forceps delivery. This would greatly improve informed decision making for both mothers and obstetricians. *Materials and Methods:* A single-arm cohort study was conducted from January 2012 to December 2016 among 49 women and their children born via forceps delivery at the Clinic for Gynecology and Obstetrics, University Clinical Center of Serbia. The Sarnat and Sarnat classification was used to evaluate the neurological status of neonates, and logistic regression analysis was employed to explore the association with perinatal factors. Long-term neurological outcomes were assessed using the Griffiths Mental Development Scale and a questionnaire for parents based on the Motor and Social Development (MSD) scale, which was derived from the Bayley-III Scale. *Results:* The main indication for forceps delivery was maternal exhaustion (79.6%), followed by fetal distress (20.4%). A pathological neurological status was observed in 16.3% of newborns, with pathological ultrasound of the CNS in 3%. A statistically significant association was observed with the Apgar score, with an odds ratio of 0.575 (95% CI: 0.407–0.813, *p* = 0.002) and perinatal asphyxia, with an odds ratio of 9.882 (95% CI: 1.111–87.902, *p* = 0.04). However, these associations were unlikely to be related to the mode of delivery. Long-term adverse neurological outcomes were seen in three cases, which accounts for 6.4%. These included mild disorders such as delayed milestone, speech delay, and motor clumsiness. *Conclusions:* The present study highlights the safety of forceps delivery regarding children’s neurological outcomes at 7 years of age. This is an important contribution to the modern management of labor, especially in light of increasing rates of cesarean deliveries worldwide.

## 1. Introduction

Assisted vaginal birth (AVB) rates have fallen globally in the last three decades, especially with the rising cesarean section (CS) rates, which have significantly increased from around 7% in 1990 to 21% today, surpassing the ideal acceptable CS rate, which is around 10–15% according to the World Health Organization (WHO) [1]. In contrast to vacuum extraction, forceps delivery is associated with reduced fetal trauma, such as an intracranial bleeding, and a greater likelihood of successful vaginal birth, but also with an increased likelihood of an obstetric anal sphincter injury (OASI). When comparing low-cavity forceps to any vacuum extractions, the protective effect of vacuum extraction, regarding OASI, was diminished [2,3,4,5]. However, a recent study reported that forceps delivery was associated with intracranial hemorrhage (RR 16.4, 95% CI 10.1–26.6), while vacuum extraction was associated with epicranial subaponeurotic hemorrhage (RR 27.5, 95% CI 20.8–36.4), when compared with spontaneous delivery [6]. It is known that intracranial hemorrhage in neonates often lead to serious adverse neurodevelopmental outcomes [7]. Moreover, there were two studies that found decreased IQ in children born with the use of forceps [8]. Thus, the examination of neurological consequences is of vital importance. On the other hand, cesarean delivery at full dilatation is associated with higher maternal morbidity and neonatal special care unit admission rates than after AVB [9]. Epidemiological studies have also reported that cesarean delivery is correlated with a higher risk of developing asthma, food allergies, type 1 diabetes, and obesity in offspring, which leads to other non-communicable diseases [10]. It also has implications for future pregnancies. There is an increased risk for preterm birth and an increased risk of unexplained fetal death in subsequent pregnancies [11,12]. The risk of an invasively adherent placenta is greater with an increasing number of cesarean deliveries, increasing from 4% with no history of cesarean delivery to 50–67% when there have been three or more cesarean deliveries [13,14,15]. Another condition associated with an increased risk of maternal and neonatal complications is impacted fetal head with a cesarean delivery [16,17]. The risk of impacted fetal head is increased in the second stage of labor and can be minimized with AVB [18].

According to systematic reviews of long-term follow-ups, cesarean deliveries do protect against long-term pelvic floor dysfunction, but there was no significant difference in this outcome between assisted and unassisted vaginal births [19,20,21].

In a recent systematic review of women and their partners’ views, emergency cesarean delivery was rated lowest by women in high-income countries, and AVB was an acceptable alternative [22].

One study that compared the outcomes of vacuum and forceps deliveries concluded that the use of forceps was associated with fewer adverse outcomes, increased quality-adjusted life years, and was more cost-effective than vacuum extraction [23].

Several potential reasons account for the underuse of AVB, especially in middle- and low-income countries. These include a lack of equipment, insufficiently trained staff, inadequate supervision, and fear of adverse outcomes [24].

In order to perform an AVB, there are maternal and fetal indications, as well as contraindications that must be followed. The fetal indication is potential fetal compromise when the vertex is well below the ischial spines. Maternal indications include maternal exhaustion and a prolonged second stage of labor (nulliparous: 3 h with epidural anesthesia and 2 h without, multiparous: 2 h with epidural anesthesia and 1 h without). Also, criteria that are necessary before proceeding with AVB are: fully dilated cervix, rupture of membranes, engaged fetal head, knowledge of the fetal position and estimated fetal weight, maternal pelvis adequate for vaginal delivery, an empty maternal bladder, and a backup plan in case the operative vaginal delivery method fails [25]. Prior to any procedure, the risks and benefits have to be thoroughly explained, and maternal consent must be obtained. It is of utmost importance that operator has the appropriate knowledge, skills, and experience and that a neonatologist is present in case of neonatal resuscitation [26].

Although a large number of studies have demonstrated the short-term safety and benefits of using forceps in the second stage of labor when indicated, data on the long-term effects on neurodevelopment in infants are sparse, especially in the Serbian population. Thus, the aim of the present study was to evaluate the neurodevelopment of children up to the age of seven, since this would improve the management of labor and informed decision making for both mothers and obstetricians in our country. The age of seven was selected because children start school at this age, and they exhibit significant growth in their mental abilities at that point. Thus, this examination provides more information about their development.

## 2. Materials and Methods

**Figure 1:**
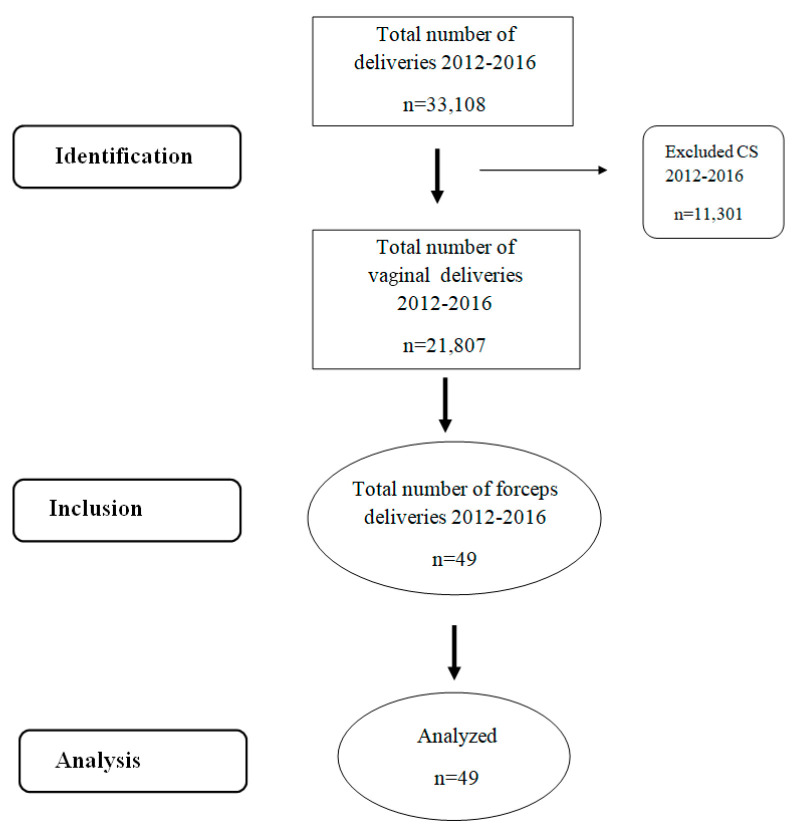
STROBE flow chart.

A single-arm cohort study was conducted from January 2012 to December 2016 among 49 women and their children born via forceps delivery at the Clinic for Gynecology and Obstetrics, University Clinical Center of Serbia. During the examined period, the total number of deliveries at the Clinic for Gynecology and Obstetrics, University Clinical Center of Serbia, was 33,108, of which 11,301 were CSs and 21,807 were vaginal deliveries, including 49 forceps deliveries. All forceps deliveries during this period were included in the study. There were no fetal anomalies or genetic disorders, and all fetuses were full-term. All labors were stimulated with oxytocin, commencing at the first stage of labor. It is known that routinely commencing oxytocin at the second stage of labor is not recommended, and the discontinuation of oxytocin stimulation in the active phase of labor does not lead to differences in the AVB rates [23]. Deliveries were performed by an experienced obstetrician after written informed consent was obtained from the mothers. The present study adhered to the STROBE guidelines (Figure 1).

The calculated power of the study was 100%. This calculation was based on our data indicating a 6.4% rate of mild neurological impairment at the age of 7, and a study conducted in one preschool institution in Serbia with 120 children from the general population aged 5 to 7 years, where the percentage of children with minor neurological dysfunction (MND) was 44.2%, of which 40.8% were classified as simple MND [27]. The calculation was performed using EZR software (version 1.67) [28]. The sample size was not taken into consideration since we included all forceps deliveries from our institution during the five years.

The evaluated parameters included maternal demographic data such as age; parity and gestational age at delivery; the duration of the first and second stages of labor, including the second stage alone (as one of the indications for forceps delivery); maternal comorbidities (gestational hypertension, gestational diabetes, and hypothyreosis); the use of epidural anesthesia; and birth injuries to the mother. The latter was evaluated by an experienced obstetrician who performed the forceps delivery and comprises cervical and vaginal tears, as well as first- and second-degree perineal tears. Third- and fourth-degree perineal tears, known as obstetric anal sphincter injuries (OASIs), were not found. Fetal parameters included birthweight; predictors of fetal distress, which are non-reassuring or abnormal cardiotocography and meconium-stained amniotic fluid; the presence of cephalohematoma; and signs of perinatal asphyxia, i.e., at least one of the following: laboratory parameters with a pH < 7 or BE ≤−12 mmol/L in the umbilical artery or in the neonate’s blood in the first hour of life, resuscitation measures applied due to slow postnatal adaptation, and/or AS < 7 in the 5th minute, as well as bilirubin levels in the newborn, i.e., the application of phototherapy [29,30].

Neonatal neurological status was classified as normal or pathological based on the newborn’s medical records—neurological examination and ultrasound of the central nervous system (CNS). A pathological neurological finding included characteristic signs of neonatal encephalopathy, which are altered mental status (e.g., irritability, decreased responsiveness, coma), seizures, hypotonia, abnormal primitive reflexes, apnea, feeding disturbance, and abnormal crying [31]. Regarding the ultrasound, ischemic encephalopathy (HIE) and intraventricular hemorrhage (IVH) grades II and III were considered pathological findings. The Sarnat and Sarnat classification was used to evaluate the degree of HIE [32].

The neurodevelopment of children up to 7 years of age was analyzed by an experienced pediatric neurologist at the Clinic for Neurology and Psychiatry for Children and Youth, University Clinical Center of Serbia, using the Griffiths Mental Development Scale. The evaluated parameters included locomotor and personal/social abilities, language, hand–eye coordination, performance, and practical reasoning [33]. At the same time, a standardized developmental questionnaire based on the Motor and Social Development (MSD) scale was completed by parents regarding whether the child had achieved certain developmental milestones. The MSD was derived from the Bayley-III scale, which is one of the standard measures of child development [34].

The presentation of the study results was approved by the Ethics Committee of the University Clinical Center of Serbia (no. 305/7) on 29 June 2023. All participants provided written informed consent for obtaining and publishing data after being given detailed information about the goals and methods of the research.

### Statistical Method

Descriptive statistics were calculated for the baseline demographic and clinical features. Continuous variables are presented as means with standard deviations, while categorical variables are presented with numbers and percentages. The normality of distribution for continuous variables was tested using the Shapiro–Wilk test. This study employed logistic regression analysis to explore the association between perinatal parameters and neurological consequences, in order to determine whether these outcomes were related to or could be attributed to the mode of delivery. The dependent variable was neurological status, and independent variables included gender, birthweight, Apgar score, parity, duration of labor, duration of the second phase of labor, episiotomy, birth injuries to the mother, intrapartum CTG, cephalhematoma, and signs of perinatal asphyxia. The results are reported as unadjusted odds ratios with 95% CI. The level of significance was set at 0.05. Statistical analysis was performed using the IBM SPSS 21 (Chicago, IL, USA, 2012) package.

## 3. Results

The average maternal age in the study was 30 ± 5.5 years. The majority of the pregnant women (81.6%) did not have any pre-existing maternal diseases. A small percentage (6.1%) had a diagnosis of gestational hypertension. Approximately 8.2% of the women were diagnosed with gestational diabetes. A minority (4.1%) had hypothyroidism as a pre-existing condition. Maternal exhaustion was the main indication (79.6%) for forceps delivery, whereas the rest (20.4%) were due to fetal distress, as indicated by cardiotocography and the characteristics of the amniotic fluid.

The mean duration of labor was 467.4 min, with the second phase lasting 69.7 min on average. An episiotomy was performed in 77.6% of cases, and rupture of the cervix, perineum, and/or vagina occurred in 40.8% of women. Epidural anesthesia was administered to 34.7% of the pregnant women, whereas the rest received perineal analgesia.

Male newborns accounted for 79.6% of the cohort, and females for 20.4%. The majority of the babies were firstborns (83.7%), all born at term, predominantly in the 40th gestational week (95.9%). Non-reassuring intrapartum CTG and meconium-stained amniotic fluid were found in 12.2% and 8.2% of cases, respectively.

Signs of perinatal asphyxia were observed in 51% of newborns, with pathological ultrasound findings of the CNS in 3% of cases. Adverse neurological status was identified in 16.3% of newborns, with cephalhematoma observed in 40.8% and hyperbilirubinemia in 34.7% of cases. Mild neurological impairment up to 7 years of age was found in three cases (6.4%). One child was reported with a delayed milestone, another had a speech delay, whereas the third exhibited motor clumsiness, speech delay, and aggressiveness. The results are presented in Table 1.

Logistic regression analysis was used to examine the association between various perinatal parameters and the adverse neurological status of newborns.

The odds ratio for gender was 0.508 (95% CI: 0.055–4.686, *p* = 0.55), indicating no statistically significant association between gender and neurological status. Birthweight showed no significant association with neurological status (OR = 1, 95% CI: 0.998–1.001, *p* = 0.708). A statistically significant association was observed with Apgar score OR 0.575 (95% CI: 0.407–0.813, *p* = 0.002), suggesting that lower Apgar scores are associated with an increased likelihood of adverse neurological outcomes. The odds ratio for parity was 0.605 (95% CI: 0.109–3.347, *p* = 0.565), indicating no significant association with neurological status. There was no significant association between either the duration of labor or the duration of the second phase of labor and neurological status (OR = 1.001, 95% CI: 0.996–1.005, *p* = 0.757) (OR = 1.002, 95% CI: 0.981–1.023, *p* = 0.847), respectively. The odds ratio for episiotomy was 0.206 (95% CI: 0.041–1.027, *p* = 0.054), and for the rupture of the cervix, perineum, and/or vagina, it was 0.426 (95% CI: 0.077–2.367, *p* = 0.329), suggesting no association between these parameters and short-term adverse neurological outcomes.

The odds ratio for intrapartum CTG was 3.083 (95% CI: 0.459–20.697, *p* = 0.246), indicating no significant relationship. The same was observed for cephalhematoma (OR = 1.562, 95% CI: 0.341–7.154, *p* = 0.565). A statistically significant association was found with signs of perinatal asphyxia, with an odds ratio of 9.882 (95% CI: 1.111–87.902, *p* = 0.04), indicating a strong correlation between signs of perinatal asphyxia and adverse neurological outcomes in newborns. It was shown that epidural anesthesia did not influence the neurological outcome of the newborns (OR = 1.157, 95% CI: 0.213–5.443, *p* = 0.88). The results are presented in Table 2.

Lower Apgar scores and signs of perinatal asphyxia are the only parameters correlated with short-term adverse neurological outcomes; however, these are unlikely to be related to the mode of delivery. Given the small number of long-term adverse outcomes, the data pertaining to these outcomes are descriptive in nature.

## 4. Discussion

In the present study, we examined the short- and long-term neurological outcomes of children born via forceps deliveries, as the number has decreased globally. Indeed, they account for only 1.1% of vaginal deliveries, according to a retrospective cohort study involving more than 22 million vaginal deliveries [35], whereas overall instrumental vaginal delivery rates range between 10% and 12% [36]. The forceps delivery rate at the Clinic for Gynecology and Obstetrics, University Clinical Center of Serbia, in 2012 and 2013 was 0.3%; in 2014 and 2015, it was 0.1%; and in 2016, it was 0.2%. In contrast, the CS rates were much higher than recommended by the WHO: in 2012, the rate was 34.28%; in 2013, it was 31.07%; in 2014, it was 32.47%; in 2015, it was 36.85%; and in 2016, it was 36.73%.

Two different studies examining the effects of AVB reported slightly lower average maternal ages than the one observed here [37,38]. The previous study indicated the same percentage for gestational hypertension but a lower percentage of 3% for gestational diabetes. There were no prolonged labors, which is in accordance with another study which noted less than 1% of prolonged labors (>18 h) [37].

Previous studies also showed a higher number of male children born via forceps [37,38,39]. The average birthweight was similar to findings from previous studies [37,38]. The average AS was 7.2 in the 5th minute, while 24% of newborns had an AS lower than 7. One cohort study showed that 17% of children born via AVB had a 5th min AS < 7 [39], whereas another reported an average 5th min AS of 8.83 among children born via AVB [38].

Adverse neurological status was identified in 16.3% of newborns, with pathological ultrasound findings of the CNS in 3% of cases. A randomized controlled trial (RCT) comparing ventouse, forceps, and cesarean deliveries favored forceps delivery as being the least likely to cause short-term adverse neonatal neurological outcomes [40]. According to one cohort study, intraventricular hemorrhages were noted in 0.05% of forceps deliveries, 0.07% of vacuum extractions, and 0.08% of cesarean deliveries. The incidences of subdural hemorrhages in this cohort were 0.14%, 0.19%, and 0.09%, respectively [41]. Another study reported that cesarean delivery in labor led to intracranial hemorrhage in 1 of 907 births, which was significantly more than for cesarean deliveries not in labor (1 of 2750) [42].

According to a previously mentioned study conducted in a preschool institution in Serbia with 120 children from the general population aged 5 to 7 years, the percentage of children with minor neurological dysfunction (MND) was 44.2%, of which 40.8% were classified as simple MND. MNDs were classified as simple if one or two of the following domains of dysfunction were present: posture and muscle tone, reflexes, involuntary movements, coordination and balance, fine manipulative skills, associated movements, senses, and cranial nerve functions. More than half were boys (62.3%), corroborating the results from another study that showed a greater prevalence of MND in boys [27]. In our study, mild neurological impairment up to 7 years of age was found in 6.4%, all of whom were boys (4% had a speech delay, 2% had motor clumsiness, and 2% were reported with delayed milestones). The probable explanation lies in differences in the development, structure, and functioning of male and female nervous systems, due to sex hormones and genetic differences, regardless of the mode of delivery [43]. Bahl et al. published similar results in a cohort study: 8.7% of children born via AVB had problems with speech, 2.4% with coordination, and 3.9% with development [39]. Our results are in line with other studies which showed no correlation between long-term morbidity and neurodevelopment of children up to the age of 5 and the mode of delivery [44,45,46]. Ayala et al. examined differences in later childhood educational outcomes after AVB and cesarean section by comparing children’s third-grade reading and math proficiencies and reported no differences [38].

Johanson et al. noted that developmental outcomes at age 5 were similar for both forceps and ventouse births [47]. It has also been shown that children born via cesarean section and normal vaginal delivery have comparable school performance at age 8 [48].

A whole-population study conducted in Australia, examining children’s school achievement after AVB, concluded that instrumental delivery did not have an adverse effect on neurodevelopment, as measured by NAPLAN performance at age 8. However, literacy and numeracy performances were slightly better after forceps delivery compared to ventouse and cesarean delivery [37].

There are several limitations of the present study. The major one is the relatively small sample due to the extremely low forceps delivery rates during the examined period. Also, this is not a comparative study but only a single-arm cohort study, so we cannot generalize the findings. Another limitation that needs to be mentioned is a potential participant bias regarding data on the acquisition of milestones in early psychomotor development, which were obtained from parents. However, since the study was conducted in a tertiary institution, the presented results are a realistic representation of forceps delivery outcomes in our population.

## 5. Conclusions

The present study aimed to highlight the safety of forceps delivery, with a focus on long-term neurodevelopment in children up to the age of seven. We reported only three cases of mild neurodevelopmental disorders, which account for six percent of our sample. Although this is a tertiary center study, the relatively small sample size may be a possible limitation. Alongside other data in the literature showing similar results, this paper represents an important contribution to informed decision making and the appropriate management of labor for both mothers and obstetricians.

## Figures and Tables

**Table 1:** Newborn and maternal baseline characteristics.

		*n* (%)
Maternal age		30 ± 5.5
Maternal comorbidity	Without	40 (81.6)
	Gestational hypertension	3 (6.1)
	Gestational diabetes	4 (8.2)
	Hypothyreosis	2 (4.1)
Parity	1	41 (83.7)
	2	5 (10.2)
	3	2 (4.1)
	4	1 (2)
Gestational age of delivery (weeks)	39	2 (4.1)
	40	47 (95.9)
Episiotomy		38 (77.6)
Birth injuries to the mother		20 (40.8)
Epidural anesthesia		17 (34.7)
Duration of the 1st and 2nd stage of labor (min)	467.4 ± 164.1	
Duration of the 2nd stage of labor (min)	69.7 ± 35.2	
Newborn gender	Male	39 (79.6)
	Female	10 (20.4)
Birthweight (grams)		3523.3 ± 465.1
Apgar score		7.2 ± 2.4
Non-reassuring intrapartum CTG		6 (12.2)
Meconium-stained amniotic fluid		4 (8.2)
Signs of perinatal asphyxia/NICU admission		25 (51)
Pathological ultrasound of CNS		1 (3)
Pathological neurological status of the newborn		8 (16.3)
Cephalhematoma		20 (40.8)
Hyperbilirubinemia		17 (34.7)
Adverse long-term neurological outcome		3 (6.4)

**Table 2:** Logistic regression—the association between perinatal factors and the neurological status of newborns.

	OR	95% CI for OR		*p*
Gender of the child	0.508	0.055	4.686	0.55
Birthweight	1	0.998	1.001	0.708
Apgar score	0.575	0.407	0.813	0.002
Parity	0.605	0.109	3.347	0.565
Duration of the 1st and 2nd stage of labor	1.001	0.996	1.005	0.757
Duration of the 2nd stage of labor	1.002	0.981	1.023	0.847
Episiotomy	0.206	0.041	1.027	0.054
Birth injuries to the mother	0.426	0.077	2.367	0.329
Intrapartum CTG	3.083	0.459	20.697	0.246
Cephalhematoma	1.562	0.341	7.154	0.565
Signs of perinatal asphyxia	9.882	1.111	87.902	0.04
Epidural anesthesia	1.157	0.213	5.443	0.88

## Data Availability

The original contributions presented in the study are included in the article, further inquiries can be directed to the corresponding author.
